# Antiviral Activity of 4′-thioIDU and Thymidine Analogs against Orthopoxviruses

**DOI:** 10.3390/v2091968

**Published:** 2010-09-16

**Authors:** Mark N. Prichard, Earl R. Kern

**Affiliations:** Department of Pediatrics, University of Alabama School of Medicine, CHB 128, 1600 6th Ave S, Birmingham AL 35233-1711, USA; E-Mail: ekern@peds.uab.edu

**Keywords:** orthopoxvirus, antiviral, nucleoside, pyrimidine, idoxuridine, deoxyuridine, 4′-thioIDU

## Abstract

The search for effective therapies for orthopoxvirus infections has identified diverse classes of molecules with antiviral activity. Pyrimidine analogs, such as 5-iodo-2′-deoxyuridine (idoxuridine, IDU) were among the first compounds identified with antiviral activity against a number of orthopoxviruses and have been reported to be active both *in vitro* and in animal models of infection. More recently, additional analogs have been reported to have improved antiviral activity against orthopoxviruses including several derivatives of deoxyuridine with large substituents in the 5 position, as well as analogs with modifications in the deoxyribose moiety including (north)-methanocarbathymidine, and 5-iodo-4′-thio-2′-deoxyuridine (4′-thioIDU). The latter molecule has proven to have good antiviral activity against the orthopoxviruses both *in vitro* and *in vivo* and has the potential to be an effective therapy in humans.

## 1. Introduction: Activity of Thymidine Analogs against the Orthopoxviruses

The efficacy of idoxuridine (IDU) against vaccinia virus replication both *in vitro* and *in vivo* was reported almost 50 years ago and helped set the stage for the development of effective antiviral therapies [1,2]. Descriptions of its efficacy in mice infected with this virus [3,4], coupled with reports of its incorporation into viral DNA [5], led to an appreciation of how the molecule might exert specific antiviral effects and thus control viral infections. But, subsequent studies with related analogs, such as 5-iodo-5′-amino-2′,5′,dideoxyuridine, showed that its phosphorylation induced by infection with herpes simplex virus (HSV) remarkably improved efficacy [6], and implicated the viral encoded thymidine kinase (TK) as the enzyme that mediated this effect [7]. While this strategy was used to enhance efficacy against HSV with E-5-(2-iodovinyl)-2′-deoxyuridine [8], which led to the development of (E)-5-(2-bromovinyl)-2′-deoxyuridine (BVDU) [9], and acyclovir [10], this approach was essentially ineffective against vaccinia virus because of the biological differences in the TK homologs encoded by the herpesviruses and the orthopoxviruses.

The resurgence of interest in therapies for orthopoxvirus infections prompted a reexamination of the antiviral activity of many agents against these viruses including IDU [11,12]. The *in vitro* efficacy of IDU was confirmed against vaccinia and cowpox viruses and was similar to that of many other 5-substituted 2′-deoxyuridine analogs [13]. Administration of IDU was also shown to delay mortality of severe combined immune deficiency (SCID) mice infected with vaccinia virus and reduced tail lesion severity in immunocompetent animals [14], which was consistent with previously published data obtained using the tail lesion model [15]. These results indicated that this class of molecule was indeed effective against the orthopoxviruses, albeit at doses much higher than used to inhibit the replication of HSV. This was not surprising since most analogs were selected for their efficacy against the herpesviruses, but it inspired the search for related molecules with the potential for improved efficacy against the orthopoxviruses.

**Figure 1.**
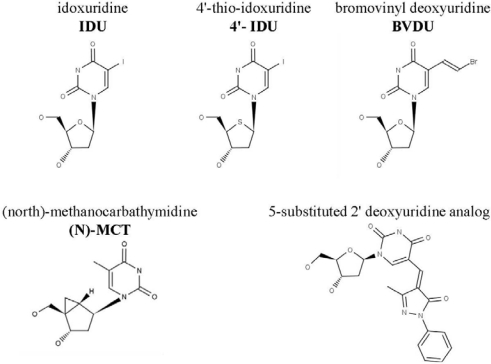
Structure of selected thymidine analogs. Structures of thymidine analogs are shown with abbreviations in bold text. The specific example of a 5-substituted deoxyuridine analog is 1-(2-deoxypentofuranosyl)-5-(3-methyl-5-oxo-1-phenyl-4,5-dihydro-4Hpyrazol-4-ylidene)pyrimidine-2,4(1H,3H)-dione [17].

The activity of these analogs against the orthopoxviruses was reviewed recently [12], however recent efforts to identify compounds with antiviral activity against vaccinia virus have identified a number of additional analogs with good activity (Figure 1). The determination of the structure of the J2 TK [16], together with genetic and enzymatic studies confirmed that this enzyme expressed by vaccinia virus might be capable of phosphorylating a wider variety of thymidine analogs than previously thought, and was unanticipated [17]. N-methanocarbathymidine ((N)-MCT), which is active against some herpesviruses, also has good activity against the orthopoxviruses both *in vitro* and *in vivo* [18]. Several novel thymidine analogs from the laboratory of Paul Torrence (Northern Arizona University, Flagstaff, Arizona) also appeared to be good inhibitors of orthopoxvirus replication in cell culture [19–22]. The 4′-thio derivative of IDU (4′-thioIDU) has also been reported to have good antiviral activity against some herpesviruses [23,24], and recently has been shown to have excellent antiviral activity against the orthopoxviruses [25]. Related 6-azathymidine-4′-thionucleosides also exhibited activity against vaccinia virus, but did not appear to require TK for their mechanism of action [26]. These data were significant because it appeared that the modification of both the heterocycle as well as the deoxyribose sugar were tolerated and had the potential to enhance the antiviral activity against the orthopoxviruses. Thus, additional efforts exploring this series of molecules promise to identify compounds that interact with unique molecular targets of the orthopoxviruses and inhibit their replication. The complex phosphorylation pathways in orthopoxvirus infected cells offer opportunities to take advantage of the selective phosphorylation of these compounds and further reduce their toxicity. Recent developments with this class of compounds will be discussed to identify common themes and potential areas of research.

## 2. Molecular Targets of Thymidine Analogs in the Orthopoxviruses

Most compounds with antiviral activity against the orthopoxviruses are inhibitors of the DNA polymerase. Mutations that impart resistance to these compounds typically map to the E9L DNA polymerase gene and cluster in conserved domains [27]. Although mutations in this gene can confer resistance to compounds like cidofovir (CDV), they also inhibit viral replication in some cell lines and in animal models of infection [28–30]. Yet, large DNA viruses also encode a host of metabolic enzymes that alter the metabolism of nucleotides to promote the replication of the viral genome. The orthopoxviruses express several such enzymes and their role in viral replication and potential for targeting by antiviral drugs have been recently reviewed [31,32]. Four viral enzymes appear to be involved in phosphorylation and stability of deoxynucleoside and deoxynucleotide analogs.

Cells infected with vaccinia virus express a unique TK that can phosphorylate thymidine [33], and appears to influence the antiviral activity of a number of compounds [34]. This enzyme is encoded by the J2R gene and homologs are encoded by all the human orthopoxviruses [35]. These enzymes belong to the type II class of TKs and are closely related to the human cellular cytosolic TK (TK1) [36,37]. Like the host kinase, these homotetrameric enzymes phosphorylate a rather narrow range of substrates and are allosterically regulated by both thymidine diphosphate and thymidine triphosphate (dTTP) [38,39]. Genetic studies with TK negative mutants of vaccinia and cowpox viruses have identified a number of inhibitors with reduced efficacy in the absence of this gene, suggesting that they are substrates for the enzyme [34,40,41]. Additionally, mutations that confer resistance to 4′-thioIDU map to this gene [42]. Enzymatic studies also confirmed that the substrate specificity of the viral enzyme is reduced compared with TK1 and many more nucleoside analogs are efficiently phosphorylated by this enzyme [16,17].

**Figure 2.**
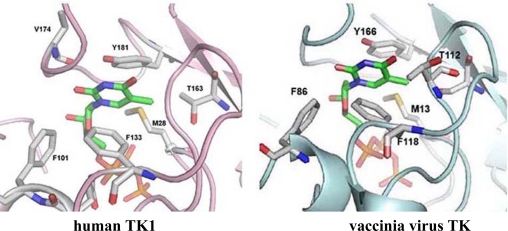
Structure of the active sites of human TK1 (left) and vaccinia virus TK (right). Both the human and vaccinia virus enzymes co-purified with the allosteric effector, dTTP, which was resolved in the published three–dimensional structures of the enzymes. Shown is dTTP bound to the active site of both the human [43], and the viral enzyme [16]. (Figure provided by Debasish Chattopadhyay, University of Alabama at Birmingham).

The structure of vaccinia virus TK in a complex with dTTP has been determined, and its substrate binding pocket appears to be slightly larger than that of the cellular enzyme such that it might accommodate slightly larger molecules [16]. Although this would be consistent with the current pharmacologic, genetic and enzymatic evidence, the crystallization of the allosteric effector in the active site of both the host and viral enzyme make it exceedingly difficult to utilize structural information in the design of new molecules (Figure 2). However, if the viral enzyme could be co-crystallized with other substrates it might provide valuable data to help understand differences in substrate specificity.

The human orthopoxviruses also encode a thymidylate kinase (TMPK) that further phosphorylates thymidine monophosphate to the level of the diphosphate [44]. The structure of this enzyme has also been determined and is similar to that of the host homolog, although the association of the dimers results in a somewhat larger active site, which permits larger substrates such as BVDU monophosphate, which has been co-crystallized in the active site [45]. Enzymatic studies in this report also confirm the phosphorylation of this substrate by the enzyme and show that it can also play a role in the phosphorylation of antiviral drugs in infected cells. The enzyme also appears to phosphorylate deoxyguanosine monophosphate and related analogs, which are not substrates of the cellular TMPK [46,47]. Vaccinia virus also encodes DNA sequences that share homology with the cellular dGMP kinase gene and it has been hypothesized to possess this enzymatic activity [48]. However, the viral gene appears to be disrupted in all isolates of vaccinia virus examined to date and thus probably does not represent a legitimate viral gene.

The fourth gene of interest is the deoxyuridine triphosphatase (dUTPase) gene (F2L) that presumably minimizes the incorporation of dUTP into viral DNA [49–50]. This enzyme is not required for viral replication *in vitro* or in mice and its potential function in viral replication is unclear, except that it is predicted to be involved in the modulation of pyrimidine metabolites [51]. Recombinant viruses that do not express this protein do not appear to have altered susceptibility to IDU, although they do appear to be modestly hypersensitive to (N)-MCT. The significance of the latter observation is unclear given uncertainties of the effect of the enzyme on nucleotide pools and does not appear to shed light on the mechanism of action of the compound.

**Figure 3.**
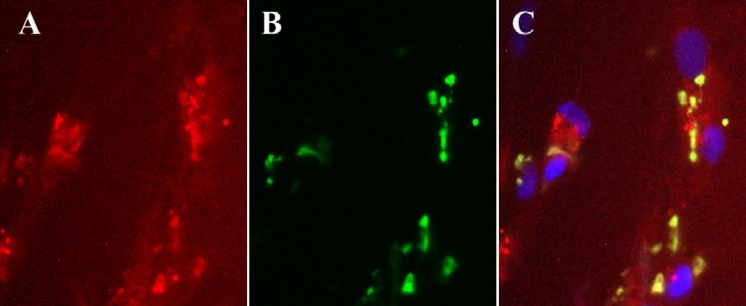
BrdU is incorporated into viral DNA within virus factories. **(A)** Cells infected with vaccinia virus were labeled with a virus-specific monoclonal antibody (red staining); **(B)** BrdU incorporated into viral DNA was visualized with a monoclonal antibody and localized to virus factories (green staining); **(C)** A merged image shown with nuclei labeled with DAPI show that the compound appears to be incorporated preferentially into viral DNA rather than in host DNA in the nuclei.

All of these enzymes have the potential to impact the antiviral activity of pyrimidine analogs in infected cells by influencing the formation of triphosphate metabolites that inhibit the viral DNA polymerase. Although it is possible to utilize this altered pathway of metabolism for the purposes of improving the selectivity of pyrimidine analogs, the pathways are incompletely understood. Understanding the impact of these viral enzymes on the antiviral activity on pyrimidine analogs is further complicated by host enzymes with partially overlapping substrate specificities, such as TK1 and the mitochondrial TK, which also phosphorylate IDU analogs. Since they are differentially expressed in the cell cycle, their impact on antiviral activity varies depending on the replication state of cell substrates [52], and perhaps the species from which the cells were derived [53]. Additionally, since viral DNA is synthesized in the cytoplasm, it is also possible that the formation of metabolites occurs within virus factories resulting in their sequestration in this compartment limiting their availability to the viral DNA polymerase in the synthesis of viral DNA. In fact, the specific incorporation of 5-bromodeoxyuridine (BrdU) into viral DNA was observed in infected cells utilizing a monoclonal antibody specific for the analog in the context of its incorporation into DNA (Figure 3). Complexities resulting from each of these factors, as well as specific binding of metabolites to the DNA polymerase can confound efforts to identify the best analogs in a series. For example, BVDU is a substrate of the vaccinia virus TK [39], and its monophosphate is further phosphorylated by the thymidylate kinase [45], yet the molecule is not particularly active *in vitro* [11,54]. Additional studies will be required to improve our understanding of this system and results from new active analogs promises to further this process.

## 3. Antiviral Activity and Mechanism of Action of (N)-MCT

The thymidine analog, (N)-MCT, is a conformationally locked nucleoside analog with an (N)-methanocarba modification in the deoxyribose portion of the molecule and its synthesis and biological activity was reviewed recently [18]. This interesting molecule serves to illustrate the complexities of metabolism and interactions with molecular targets that result in antiviral activity. This compound exhibits antiviral activity against the alphaherpesviruses and appears to derive some of its specificity through selective phosphorylation by the TK homologs encoded by this group of viruses [55]. Although both (N)-MCT and the (South)-methanocarbathymidine analog, (S)-MCT, are substrates for herpes simplex virus type 1 (HSV-1) TK, (S)-MCT appears to be a better substrate for the enzyme, which was confirmed in co-crystallization studies that showed that the north conformation of the nucleobase in (N)-MCT induces a shift in isoleucine 197 of the enzyme relative to that of thymidine. A specific inhibitor of the HSV-1 TK (R0-32-2313) [56], was also used in further studies that suggested the phosphorylation pathways for (N)-MCT were complicated and also likely involved other cellular kinases [57]. Specific inhibition of the HSV-1 TK inhibited the formation of (N)-MCT diphosphate indicating that the thymidylate kinase activity associated with this enzyme was involved with the formation of this metabolite [58]. However, levels of the monophosphate were unaffected by the TK inhibitor suggesting that cellular kinases can also phosphorylate (N)-MCT. It is unclear which cellular enzymes might also participate in its phosphorylation however, since the compound is a poor substrate for the human cytosolic TK1 [59].

It is interesting that (N)-MCT is an inhibitor of HSV-1 and HSV-2 replication, while (S)-MCT is essentially inactive [60]. This result is consistent with studies in murine MC38 cells expressing the HSV-1 TK, where (N)-MCT is incorporated into cellular DNA by the cellular DNA polymerases at much higher levels than (S)-MCT, notwithstanding the much higher levels of (S)-MCT triphosphate [61]. Thus, the specificity of the HSV TK, unidentified host kinases and presumably the viral DNA polymerase all contribute to the antiviral activity of the compound.

The orthopoxviruses are also susceptible to the action of (N)-MCT, which inhibited the *in vitro* replication of both vaccinia and cowpox viruses at concentrations less than 2 μM (Table 1) [40]. However, the antiviral activity also appeared to be cell line dependent, with the best antiviral activity seen in murine cells, with less activity in other species including cells derived from the rabbit, monkey, and humans [53]. The compound is also effective in reducing mortality in mice infected with cowpox virus and vaccinia viruses (Table 2) [40,53,62].

The mechanism of action of this compound against the orthopoxviruses has not been examined in detail but preliminary work has provided a few insights. Like IDU, the compound exhibited reduced efficacy against a TK negative isolate of cowpox virus, which suggested that the viral kinase could phosphorylate the compound [40]. These data were confirmed with enzymatic studies that indicated the molecule is a substrate for the vaccinia virus TK with a *K_m_* similar to that of thymidine. In infected cells, vaccinia virus TK appeared to significantly increase the levels of the monophosphate metabolite in infected cells although no differences were observed in the levels of the triphosphate [41]. While these data are consistent with the viral TK phosphorylating (N)-MCT, it is complicated by cellular enzymes that also appear to phosphorylate the compound. The formation of the monophosphate metabolite also appears to occur in uninfected cells such the initial phosphorylation step by either the HSV or vaccinia virus TK homologs should not be absolutely required for antiviral activity [57]. In fact, in many cell lines it has not been possible to demonstrate that the vaccinia virus TK is significantly involved in the efficacy of the compound [53]. These data clearly indicate that cellular enzymes can play a significant role in the activation of the compound. While an obvious candidate cellular enzyme is the cytosolic TK (TK1), (N)-MCT has been shown to be a poor substrate for this enzyme and its contribution toward to the conversion of the monophosphate is thought to be negligible [59]. It is also possible that the mitochondrial enzyme (TK2) might phosphorylate the compound but at this time evidence for this is lacking. The phosphorylation of the compound in uninfected cells could potentially result in toxicity, which will need to be considered as the molecule undergoes further development.

The active metabolite of (N)-MCT that inhibits orthopoxvirus replication is presumed to be the triphosphate, but there is no direct evidence that it is a substrate for the viral DNA polymerase. Significant quantities of the triphosphate are observed in infected cells, although that quantity of this metabolite does not appear to correlate either with levels of the monophosphate, or the differential efficacy observed for cell lines derived from other species [41]. A recombinant virus that does not express the dUTPase appears to be modestly hypersensitive to (N)-MCT, but this does not necessarily imply that the active form is the triphosphate since specific effects of this enzyme on pyrimidine metabolism are poorly understood [51]. While this compound inhibits viral DNA replication with an efficacy comparable to that of its EC_50_ and is likely responsible for the inhibition of viral replication, it remains possible that this is an indirect effect and it may actually inhibit enzymes other than the DNA polymerase that are required for DNA replication.

## 4. Thymidine Analogs with Large Substituents at the 5 Position

Thymidine analogs with modifications at the 5 position have also been shown to be active against replication of vaccinia virus and have been reviewed recently [12]. Most substituents in this position have been small, notably halogens, amino, nitro and vinyl groups and some of these analogs are inhibitors of thymidylate synthetase [63]. More recent studies have investigated compounds with larger moieties that appear to retain antiviral activity against vaccinia virus [19]. Several publications have resulted from the evaluation of antiviral activity of these compounds synthesized in the laboratory of Dr. Torrence [17,19–22]. Most of the compounds in this series contain large substituents, including heterocyclic moieties, and inhibited the *in vitro* replication of vaccinia virus with EC_50_ values in the low micromolar range (Table 1) [20,22]. Subsequent studies indicated that the antiviral activity of the compounds was largely dependent on the orthopoxvirus TK [17]. All of the compounds tested proved to be good substrates for vaccinia virus TK with *K_m_* values comparable to or below that for thymidine and many were poor substrates for TK1 [17]. Thus, the larger binding pocket observed in vaccinia virus TK appears to accommodate the large substituents at the 5 position of deoxyuridine. The ultimate molecular target of these molecules is presumed to be the viral DNA polymerase, but has not been investigated.

Although this series of compounds exhibited good antiviral activity *in vitro*, it did not translate to *in vivo* activity since it was unable to reduce mortality in mice infected with vaccinia virus [64]. One of the compounds was shown to be degraded rapidly by porcine liver esterase [20], raising the possibility that poor pharmacokinetics are related to its lack of antiviral activity in animals.

## 5. Inhibition of Orthopoxvirus Replication with 4′-Thio Pyrimidine Analogs

Successes with (N)-MCT prompted an evaluation of additional thymidine analogs with modifications in the deoxyribose sugar. A series of 4′-thiopyrimidine analogs was synthesized and their antiviral activity against the alphaherpesviruses was reported previously [24]. Further studies evaluated a similar series against the alphaherpesviruses as well as cytomegalovirus (CMV) and some analogs, including 4′-thioIDU exhibited some activity against all the viruses [23]. The activity of this compound was subsequently evaluated against all the human herpesviruses including TK negative isolates of HSV and UL97 deficient isolates of CMV and showed that antiviral activity was dependent on the HSV TK homolog, but not the UL97 kinase in CMV [65]. Since the compound retains antiviral activity against CMV, which has no TK homolog, it appears that cellular enzymes are also capable of phosphorylating it to some degree. Consistent with this idea, EC_50_ values against TK negative strains of HSV are the same as those against CMV in primary human fibroblast cells. This report also utilized a monoclonal antibody to show that compound was incorporated into the DNA of HSV-2 and CMV and confirmed that they were substrates of the DNA polymerase. Furthermore, the incorporation into the host genome was noted in a subset of cells and confirmed that when cells were actively dividing, the compound was phosphorylated by cellular kinases and the triphosphate metabolite was a substrate for a host DNA polymerase. These results were expected and are similar to those observed with the related analogs BrdU and IDU [66].

The activity of 4′-thioIDU against the orthopoxviruses proved to be much greater than against the herpesviruses and was superior to that of IDU [25]. Several related 4′ pyrimidine analogs also had good antiviral activity (Table 1), but 4′-thioIDU proved to have the best combination of antiviral activity, low toxicity, and spectrum of antiviral activity and was subsequently selected by us for additional studies. This compound also was very active in animals infected with cowpox virus. Parenteral or oral administration of 5 mg/kg to infected mice significantly reduced mortality even if therapy was initiated 4 days after infection (Table 3), [25]. Subsequent studies also indicated that the compound was more potent than CDV and significantly reduced mortality with concentrations as low as 0.3 mg/kg [64]. This level of activity of 4′-thioIDU in mice is superior to that previously reported for IDU and (N)-MCT [14,40,67], and indicates that additional studies with this compound are warranted to determine its potential for use in treatment of orthopoxvirus infection of humans.

Further development of any molecule for the therapy of orthopoxvirus infections is dependent on its ability to inhibit the replication of virus isolates resistant to either CDV (or its prodrug, CMX001) or ST-246. Virus isolates that were resistant both drugs proved to be fully susceptible to 4′-thioIDU and confirmed that the compound possessed a mechanism of action distinct from either CDV, its lipophilic conjugated prodrug CMX001, or ST-246 (Table 4) [25]. Combinations of 4′-thioIDU together with either CMX001 or ST-246 synergistically inhibited viral replication *in vitro*, which is also consistent with each having a different mechanism of action [68]. A recombinant virus lacking the TK (VVTK::luc) also exhibited reduced susceptibility to 4′-thioIDU suggesting that it was involved in the activation of the compound [25]. This result was confirmed against TK positive and TK negative isolates of cowpox virus, and other studies in vaccinia virus that showed that 4′-thioIDU was a good inhibitor of viral DNA synthesis. Subsequent studies reported the selection of a 4′-thioIDU-resistant vaccinia virus isolate that acquired a 5 nucleotide deletion in the TK gene, resulting in a frameshift and a premature truncation following the first 51 amino acids of the protein but no mutations were observed in the DNA polymerase [42]. These data confirmed that the TK played a significant role in the mechanism of action of the compound. The resistant isolate also remained fully susceptible to both ST-246 as well as CMX001, consistent with the absence of cross resistance observed previously.

**Figure 4.**
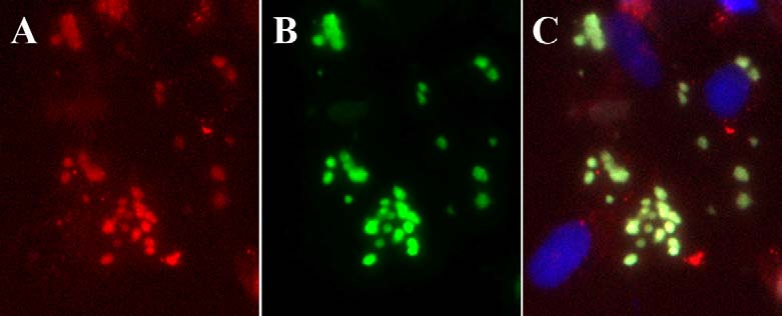
4′-thioIDU is incorporated in virus factories. **(A)** Cells infected with vaccinia virus were labeled with a monoclonal antibody (red staining); **(B)** 4′-thioIDU incorporated in viral DNA was visualized with a monoclonal antibody and localized to virus factories (green staining); **(C)** a merged image shown with nuclei labeled with DAPI show that most of the compound appears to be incorporated in viral DNA rather than in host DNA in the nuclei.

The use of monoclonal antibodies that are specific for IDU and BrdU molecules that have been incorporated into DNA also provided evidence to indicate that 4′-thioIDU is incorporated into DNA and provided a useful tool to study the mechanism of action of the compound [65]. Cells infected with vaccinia virus were exposed to 4′-thioIDU for 10 min prior to fixation, and its incorporation into DNA was visualized by immunofluorescence (Figure 4). The compound was incorporated into viral DNA within virus factories and indicated it was a substrate for the viral DNA polymerase and that its mechanism of action was likely similar to that of IDU. Furthermore, it appeared to be incorporated exclusively in viral DNA and no detectable staining of host DNA in the nucleus was observed, suggesting that it specifically targeted viral DNA synthesis and is similar to results shown with BrdU (Figure 2). However, some nuclear staining was identified in nuclei of uninfected cells which is also consistent with the phosphorylation and incorporation of the compound into host DNA during the S phase of the cell cycle (data not shown), as has been observed with BrdU and IDU [66]. It is unclear if the specific incorporation in viral DNA is a result of the selective phosphorylation and sequestration of the compound in virus factories or the inhibition of host DNA synthesis by the virus, but it illustrates the selective activity of this compound against vaccinia virus replication and is consistent with the antiviral data.

Another report also documented the modest antiviral activity of another analog, dideoxy-6-azathymidine 4′-thionucleoside [26]. This compound did not appear to exhibit TK dependence and suggested that its mechanism of action was distinct from that of 4′-thioIDU.

## 6. Conclusions

The efficacy of pyrimidine analogs against the orthopoxviruses has been known for many years but recent concerns regarding the orthopoxviruses as weapons of bioterror have resulted in renewed interest in these compounds. Such analogs have many attractive qualities, particularly, their spectrum of antiviral activity that includes many DNA viruses including both the herpesviruses and the orthopoxviruses. Although the clinical benefit from this broader activity is modest, it could provide a viable development path for the development of a compound with antiviral against the orthopoxviruses. This will be critical for any candidate compounds proceeding to the clinic since it is impossible to conduct pivotal clinical studies against the orthopoxviruses, which would be required for their approval. However, if such a compound were also active against another DNA virus, such as a herpesvirus, it would provide an alternative mechanism for conducting clinical studies for determining efficacy and toxicity in humans and might prove to be economically viable. Once approved, the emergency use of the agent could then be approved for use against variola virus or monkeypox virus infections. Additional studies with these and other analogs may also serve to identify other molecules with desirable properties that might become available to treat orthopoxvirus infections in humans.

## Figures and Tables

**Table 1.** Activity of thymidine analogs against vaccinia virus and cowpox virus^a^.

**Compound**	**Vaccinia virus (EC_50_, μM)^b^**	**Cowpox virus (EC_50_, μM)**	**Cytotoxicity (CC_50_, μM)**
cidofovir	19 ± 11	29 ± 6.1	>317 ± 0
idoxuridine	8.4 ± 3.3	3.7 ± 2.7	>100 ± 0
fialuridine	1.5 ± 0.4	0.2 ± 0.1	>100 ± 0
(N)-MCT	0.6 ± 0.1	1.5 ± 1.2	>100 ± 0
5-iodo-4′-thio-2′-deoxyuridine (4′-thioIDU)	0.5 ± 0.2	0.1 ± 0.04	>100 ± 0
1-(2-deoxy, 4′thio-β-D-ribofuranosyl)-thymidine	0.03 ± 0.01	0.02 ± 0.01	29 ± 4.0
4-thio-β-D-arabinofuranosyl)-cytidine	0.3 ± 0.2	1.6 ± 0.8	53 ± 6.4
1-(4-thio-β-D-arabinofuranosyl)-5-fluoro cytidine	0.1 ± 0.01	0.4 ± 0.1	4.6 ± 1.1
5-iodo-4-thio-3′,5′-di-O-acetyl-2′-deoxyuridine	0.9 ± 0.3	0.3 ± 0.2	>80 ± 28
5-bromo-4′-thio-2′-deoxyuridine	0.1 ± 0.02	0.05 ± 0.04	>100 ± 0
5-trifluoromethyl-2′-deoxy-4′-thiouridine	0.1 ± 0.004	0.1 ± 0.01	>100 ± 0

a Adapted from [25]

b Concentration of compound sufficient to reduce viral replication by 50% (EC_50_).

**Table 2.** Efficacy of (N)-MCT in BALB/c mice infected intranasally with vaccinia or cowpox virus^a^.

**Treatment^b^**	**Mortality**		**P-value**	**MDD^c^**	**P-value**
	**Number**	**Percent**			
**Vaccinia virus**					
vehicle	15/15	100	-	7.9	-
**CDV**					
15 mg/kg	0/15	0	<0.001	-	-
**(N)-MCT**					
50 mg/kg	0/15	0	<0.001	-	-
16.7 mg/kg	2/15	13	<0.001	7.5	NS^d^
5.6 mg/kg	12/15	80	NS	8.2	NS
**Cowpox virus**					
vehicle	15/15	100	-	9.6	-
**CDV**					
15 mg/kg	0/15	0	<0.001	-	-
**(N)-MCT**					
50 mg/kg	2/15	13	<0.001	7.5	NS
16.7 mg/kg	3/15	20	<0.001	13.3	0.01
5.6 mg/kg	6/15	40	<0.001	14.0	0.05

a Adapted from [40];

b (N)-MCT was prepared in 0.4% carboxymethylcellulose and delivered i.p. twice daily in 0.1 ml doses. CDV was prepared in sterile saline and given i.p. once daily in 0.1 ml doses. All animals were treated for 5 days beginning 24 h post infection;

c Mean Day of Death;

d Not significant.

**Table 3.** Effect of oral treatment with 4′-thioIDU on mortality of BALB/c mice inoculated intranasally with Cowpox Virus ^a^.

**Treatment^b^**	**Mortality**		**P-value**	**MDD + SD^c^**	**P-value**
	**Number**	**Percent**			
**Vehicle + 3 days**	14/15	93		11.9 ± 2.2	
**CDV + 3 days**					
15 mg/kg	0/14	0	<0.001		
**4**′**-thioIDU + 3 days**					
15 mg/kg	1/15	7	<0.001	14.0	NS^c^
5 mg/kg	0/15	0	<0.001		
1.5 mg/kg	2/15	13	<0.001	14.0 ± 5.7	NS^c^
**Vehicle + 4 days**	15/15	100		11.7 ± 2.3	NS
**CDV + 4 days**					
15 mg/kg	0/15	0	<0.001		
**4**′**-thioIDU + 4 days**					
5 mg/kg	4/15	27	<0.001	13.3 ± 1.3	<0.05
**Vehicle + 5 days**	12/15	80		14.6 ± 3.9	
**CDV + 5 days**					
15 mg/kg	4/15	27	0.01	10.5 ± 1.9	0.07
**4**′**-thioIDU + 5 days**					
5 mg/kg	13/15	87	NS^c^	9.8 ± 1.3	<0.001

a. Adapted from [25];

b. 4′-thioIDU was suspended in vehicle (10% DMSO in 0.4% CMC) and given orally in 0.2 ml doses. CDV was prepared in sterile saline and given i.p. in 0.1 ml doses. Animals were treated twice daily with vehicle or 4′-thioIDU for five days, except for CDV which was dosed once daily, beginning 3, 4 or 5 days post viral inoculation;

c. MDD = mean day of death; SD = standard deviation; NS = not significant when compared to the placebo control.

**Table 4.** Activity of 4′-thioIDU Against Resistant Mutants of Vaccinia Virus^a^.

**Compound**	**WR (EC_50_, μM)^b^**	**CDV^R^ 15 (EC_50_, μM)**	**VV911 (ST-246^R^) (EC_50_, μM)**	**VVTK::luc TK deficient (EC_50_, μM)**
4′-thioIDU	0.04 ± 0.02	0.04 ± 0.01	0.02 ± 0.003	0.3 ± 0.01
CDV	11 ± 1.5	62 ± 34	33 ± 5.0	9.4 ± 0.1
ST-246	0.07 ± 0.01	ND^c^	>20 ± 0	ND^b^
IDU	2.8 ± 0.3	1.0 ± 0.1	2.4 ± 0.4	7.1 ± 4.3

a. Adapted from [25];

b. Concentration required to reduce plaque formation by 50%. Values presented are the average of duplicate determinations with the standard deviations shown;

c. Not determined.
